# Increased oxidative stress according to number of risk factors in metabolic syndrome patients

**DOI:** 10.1186/1758-5996-7-S1-A134

**Published:** 2015-11-11

**Authors:** Danielle Venturini, Caroline Hellen Rampazzo Alves, Shirley Aparecida Fabris de Souza, Décio Sabbatini Barbosa

**Affiliations:** 1 Universidade Estadual de Londrina, Londrina, Brazil

## Background

Metabolic syndrome (MetS) comprises pathological conditions that include insulin resistance, arterial hypertension, visceral adiposity and dyslipidemia, which favors the development of cardiovascular diseases and type 2 diabetes. Advanced oxidation protein products (AOPPs) have been reported as the most appropriate parameter for determination of oxidative stress (OS) in MetS patients and are formed during oxidative stress by the action of chloraminated oxidants, mainly hypochlorous acid and chloramines, produced by myeloperoxidase in activated neutrophils.

## Aim

The objective of the present study was to correlated two biomarkers of OS with metabolic features in MetS patients.

## Materials and methods

This study evaluated 48 women, aged 32-58 yrs. recruited from University Hospital of Londrina, Paraná, Brazil. The groups were divided according to MetS components in 3 groups, G1 (with 3 components), G2 (with 4 components) and G3 (with 5 components). MetS was defined following the Adult Treatment Panel III (ATP III) criteria. After fasting for 12 h, the subjects underwent the following laboratory blood analysis: glucose, total cholesterol (TC), high density lipoprotein cholesterol (HDLc), low density lipoprotein cholesterol (LDLc), triacylglycerol (TG), uric acid and C reactive protein (CRP) which were evaluated by a biochemical auto-analyzer (Dimension Dade AR, Dade Behring, Deerfield, IL, USA), using Dade Behring kits. Advanced oxidation protein products (AOPP), as markers of protein damage, and total antioxidant capacity (TRAP) as antioxidant were evaluated by the semiautomated method described by Witko-Sarsat et al and chemiluminescence, respectively. Pro-oxidant-antioxidant imbalance (PAI) was calculated divided AOPP/TRAP.

## Results

The G3 group presented high levels of BMI, WC, serum levels of glucose, CRP, uric acid, AOPP and PAI when compared with G1, whereas TRAP was significantly lower in the G3 group when compared to G1 and G2 groups. G3 also presented high levels of glucose, CRP, AOPP and lower levels of TRAP when compared to G2 group. With regard to the relationship between oxidative stress markers and metabolic syndrome components, there were a positive correlation between AOPP and TG (r: 0,810; p: 0.0002), LDL (r: 0,630; p: 0.015) and CRP (r: 0,593; p: 0.019).

## Conclusion

This study showed that the metabolic disorders were determinant for the redox imbalance, characterized by increased plasma oxidation and reduced antioxidant capacity.

**Figure 1:**
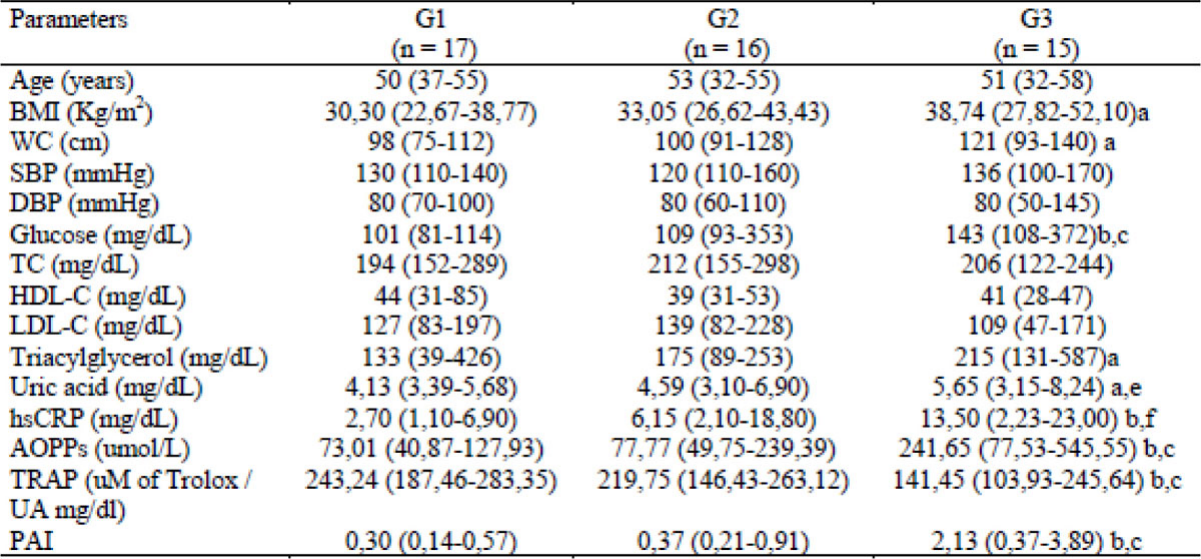
Anthropometric, biochemical and oxidative stress parameters according the number of metabolic syndrome components. Kruskal-Wallis with post test Dunn. Data are median (25%-75%). G1: subjects with 3 MetS components, G2: subjects with 4 MetS components, G3: subjects with 5 MetS components, BMI, body mass index; WC, waist circumference; SBP, systolic blood pressure; DBP, diastolic blood pressure; TC, total cholesterol; HDL-C, high density lipoprotein-cholesterol; LDL-C, low density lipoprotein-cholesterol; hsCRP: high sensitivity C-reactive protein; AOPPs, advanced oxidation protein products; TRAP, total radical trapping antioxidant parameter; UA, uric acid; PAI, pro-oxidant-antioxidant imbalance. a: p<0.05 (G3 vs G1) b: p<0.0001 (G3 vs G1) c: p<0.0001 (G3 vs G2) d: p<0.05 (G3 vs G2) e: p<0.05 (G2 vs G1) f: p<0.0001 (G3 vs G2)

